# Supplementary figures and images for: Exposure to traffic-related air pollution and bacterial diversity in the lower respiratory tract of children
Source: PLoS One. 2021 Jun 24;16(6):e0244341. doi: 10.1371/journal.pone.0244341 (PMC8224880; doi:10.1371/journal.pone.0244341)

**
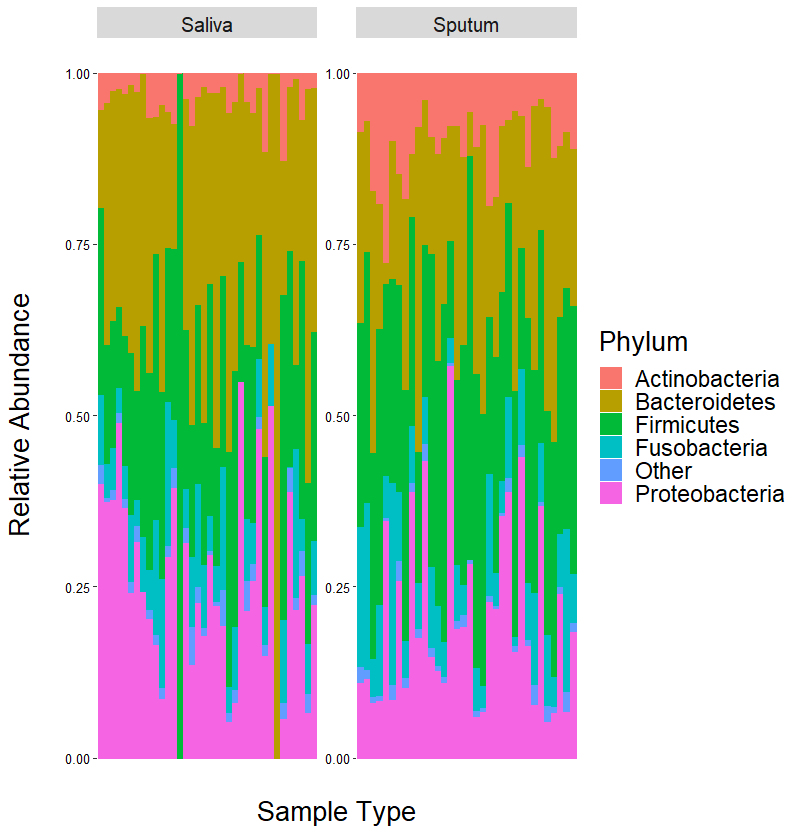
**

S1 Fig. Bar plots showing the relative abundance of each bacterial phyla in sputum and saliva.

Supplement: S1 Fig — (DOCX) [file pone.0244341.s001.docx]
