# Supplementary material for: Exposure to traffic-related air pollution and bacterial diversity in the lower respiratory tract of children
Source: PLoS One. 2021 Jun 24;16(6):e0244341. doi: 10.1371/journal.pone.0244341 (PMC8224880; doi:10.1371/journal.pone.0244341)
Supplement: S2 Fig — (DOCX) [file pone.0244341.s002.docx]

**
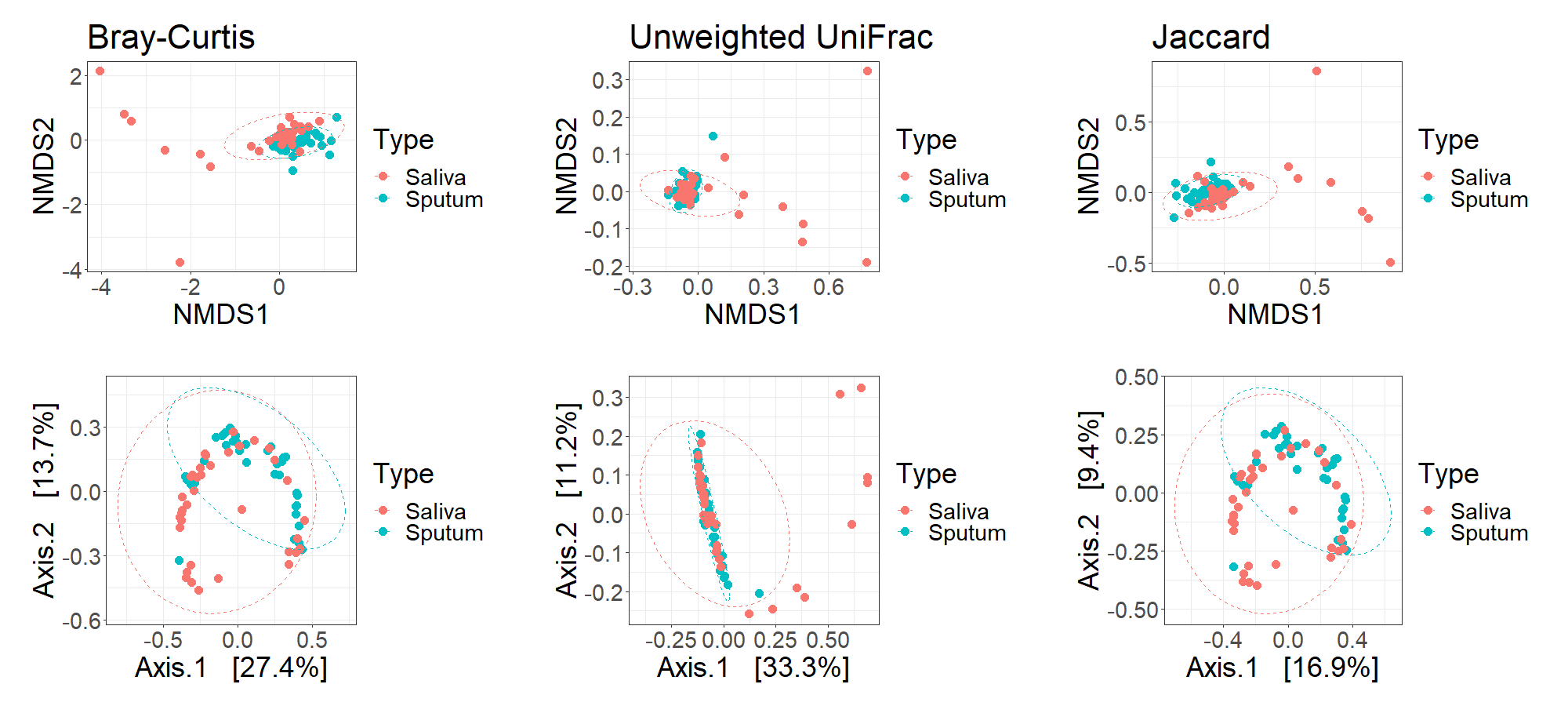
**

S2 Fig. NMDS (upper graphs) and MDS (lower graphs) plots using Bray-Curtis, unweighted UniFrac, and Jaccard beta diversity measurements to compare the bacteriomes in sputum and saliva samples
