# Supplementary material for: Exposure to traffic-related air pollution and bacterial diversity in the lower respiratory tract of children
Source: PLoS One. 2021 Jun 24;16(6):e0244341. doi: 10.1371/journal.pone.0244341 (PMC8224880; doi:10.1371/journal.pone.0244341)
Supplement: S3 Fig — Box plots comparing bacterial diversity indices in sputum between (A) asthma status and (B) gender, including Shannon diversity, number of observed amplicon sequence variants (ASVs), and Faith’s phylogenetic diversity. (DOCX) [file pone.0244341.s003.docx]

**
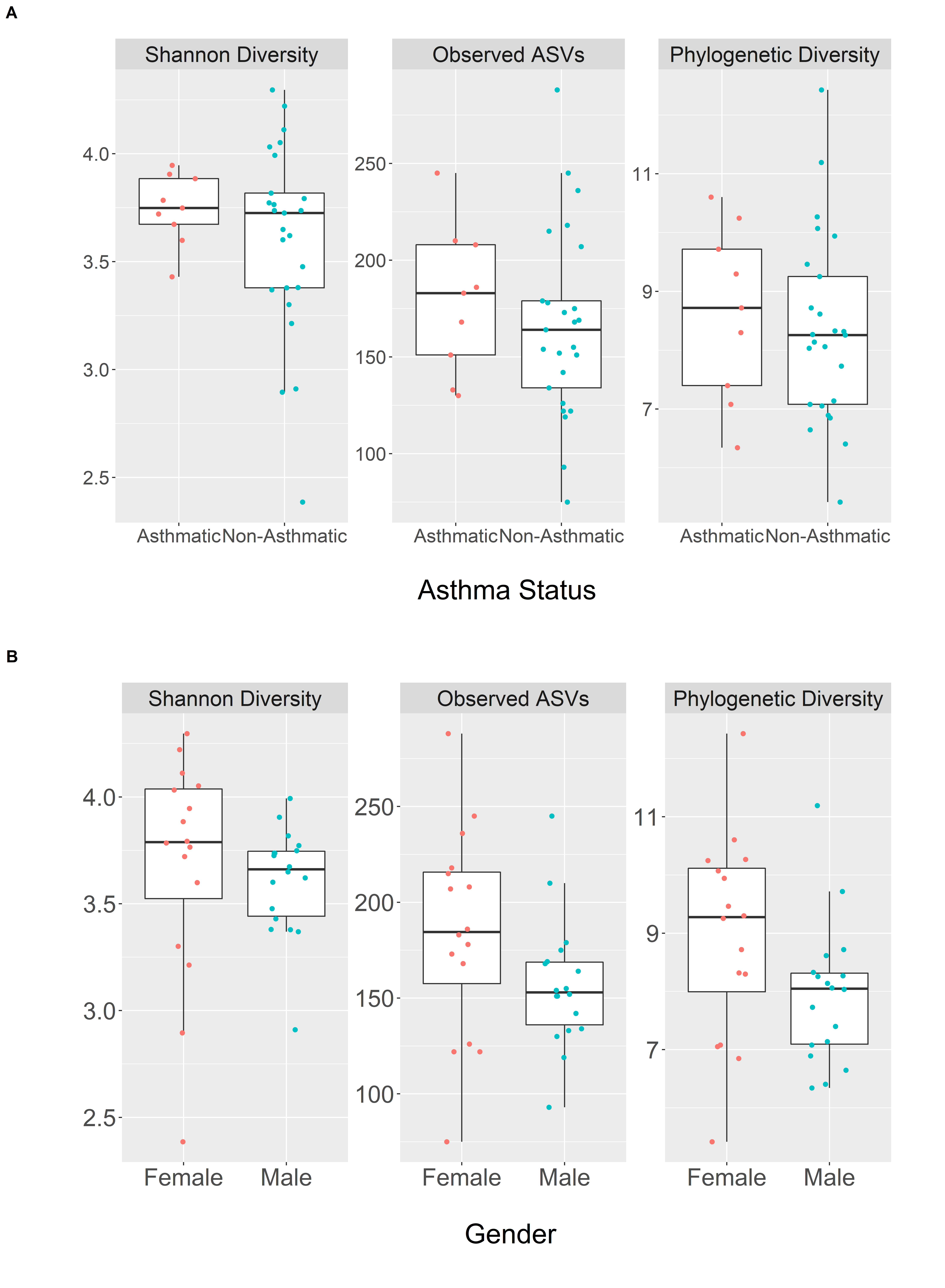
**

S3 Fig. Box plots comparing bacterial diversity indices in sputum between (A) asthma status and (B) gender, including Shannon diversity, number of observed amplicon sequence variants (ASVs), and Faith’s phylogenetic diversity.
