# Supplementary material for: Exposure to traffic-related air pollution and bacterial diversity in the lower respiratory tract of children
Source: PLoS One. 2021 Jun 24;16(6):e0244341. doi: 10.1371/journal.pone.0244341 (PMC8224880; doi:10.1371/journal.pone.0244341)
Supplement: S4 Fig — Box plots comparing bacterial diversity indices in saliva between (A) TRAP exposure groups, (B) asthma status groups, and (C) gender, including Shannon diversity, number of observed amplicon sequence variants (ASVs), and Faith’s phylogenetic diversity. (DOCX) [file pone.0244341.s004.docx]

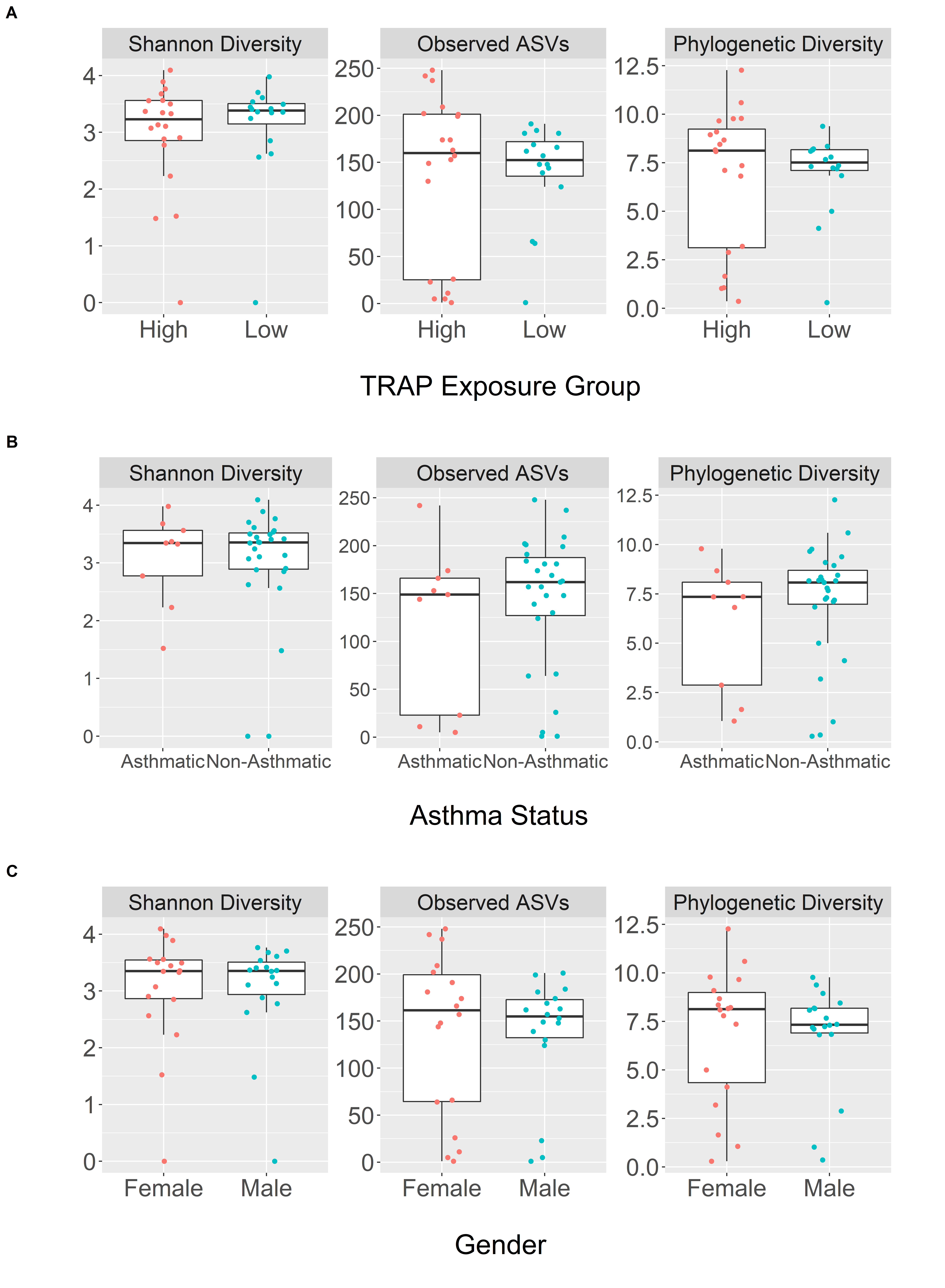


S4 Fig. Box plots comparing bacterial diversity indices in saliva between (A) TRAP exposure groups, (B) asthma status groups, and (C) gender, including Shannon diversity, number of observed amplicon sequence variants (ASVs), and Faith’s phylogenetic diversity.
