# Supplementary material for: Exposure to traffic-related air pollution and bacterial diversity in the lower respiratory tract of children
Source: PLoS One. 2021 Jun 24;16(6):e0244341. doi: 10.1371/journal.pone.0244341 (PMC8224880; doi:10.1371/journal.pone.0244341)
Supplement: S5 Fig — (DOCX) [file pone.0244341.s005.docx]

***

***

S5 Fig. Bar plots showing the relative abundance of bacterial phyla in sputum across TRAP exposure groups, asthma status groups, and genders.
