# Supplementary material for: Exposure to traffic-related air pollution and bacterial diversity in the lower respiratory tract of children
Source: PLoS One. 2021 Jun 24;16(6):e0244341. doi: 10.1371/journal.pone.0244341 (PMC8224880; doi:10.1371/journal.pone.0244341)
Supplement: S6 Fig — Each line on the y-axis indicates the family, each point represents an individual ASV within that family, and the color of the point indicates the phylum. (DOCX) [file pone.0244341.s006.docx]

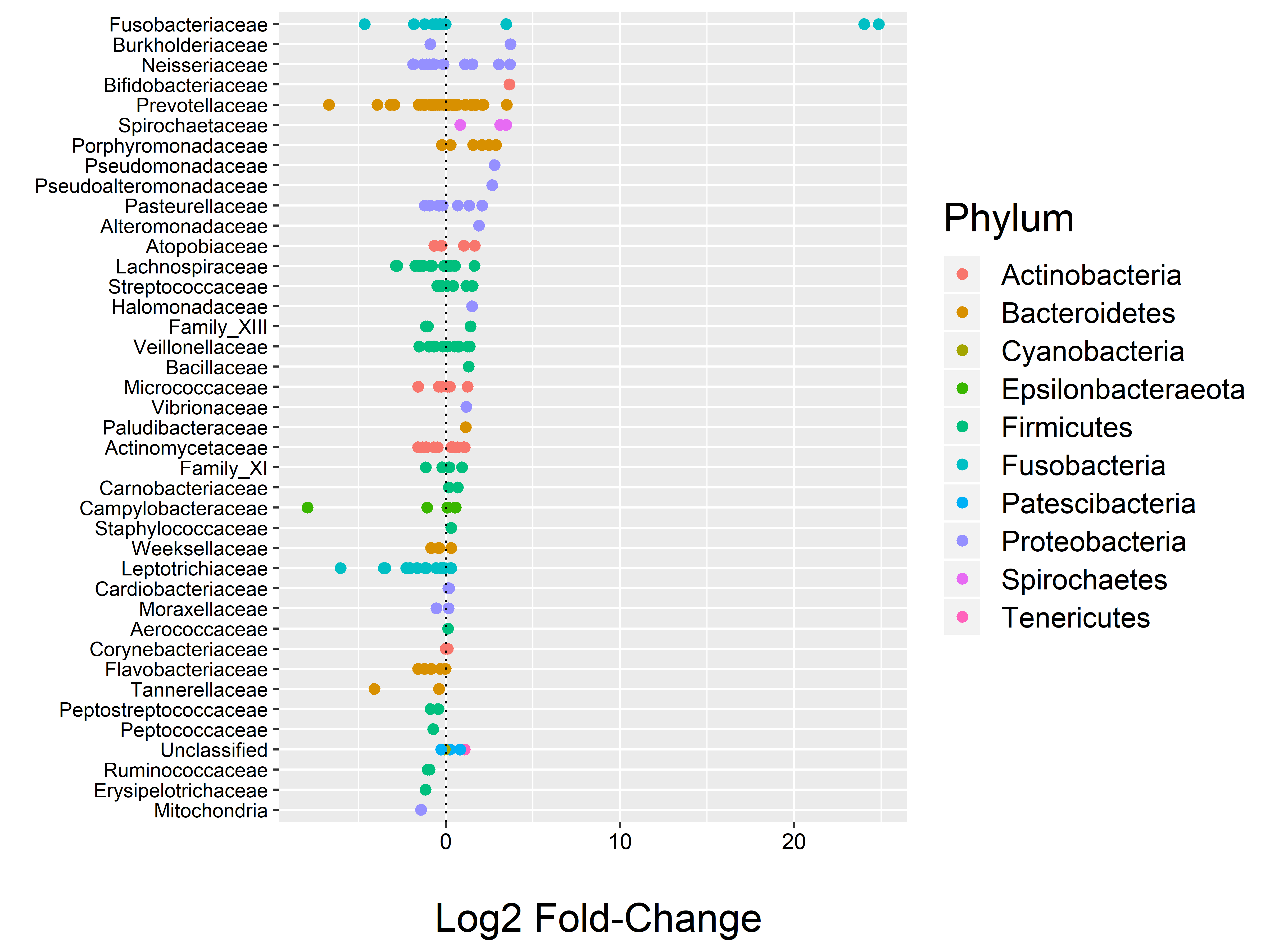


S6 Fig. DESeq2 results showing the log2 fold-change values (x-axis) in sputum bacteriome between genders. Each line on the y-axis indicates the family, each point represents an individual ASV within that family, and the color of the point indicates the phylum.
