# Supplementary material for: Exposure to traffic-related air pollution and bacterial diversity in the lower respiratory tract of children
Source: PLoS One. 2021 Jun 24;16(6):e0244341. doi: 10.1371/journal.pone.0244341 (PMC8224880; doi:10.1371/journal.pone.0244341)
Supplement: S1 Table — (DOCX) [file pone.0244341.s008.docx]

| S1 Table. Mean (95% confidence interval) and Wilcoxon rank sum test p-value for each fungal alpha diversity measure in the sputum by TRAP exposure, asthma status, and gender. | | | | | | | | | |
| --- | --- | --- | --- | --- | --- | --- | --- | --- | --- |
|  | **TRAP Exposure** | | | **Asthma Status** | | | **Gender** | | |
|  | **High** (n=5) | **Low** (n=5) | **p-value** | **Asthmatic** (n=5) | **Non-Asthmatic** (n=5) | **p-value** | **Female** (n=6) | **Male** (n=4) | **p-value** |
| **Number of Observed ASVs** | 4.40 (2.59-6.21) | 6.60 (0.64-12.6) | 1.0 | 7.00 (1.21-12.8) | 4.00 (2.25-5.75) | 0.53 | 3.50 (1.16-5.84) | 8.5 (2.18-14.8) | 0.09 |
| **Shannon Diversity** | 0.77 (0.29-1.23) | 0.79 (0.17-1.41) | 1.0 | 0.92 (0.29-1.55) | 0.64 (0.23-1.05) | 0.55 | 0.60 (0.12-1.08) | 1.05 (0.52-1.58) | 0.26 |
| **Phylogenetic Diversity** | 1.81 (1.41-2.21) | 1.35 (0.65-2.05) | 0.83 | 1.73 (1.05-2.41) | 1.43 (0.94-1.92) | 0.40 | 1.34 (0.73-1.95) | 1.94 (1.73-2.15) | 0.11 |
