# Supplementary material for: Exposure to traffic-related air pollution and bacterial diversity in the lower respiratory tract of children
Source: PLoS One. 2021 Jun 24;16(6):e0244341. doi: 10.1371/journal.pone.0244341 (PMC8224880; doi:10.1371/journal.pone.0244341)
Supplement: S1 File — (DOCX) [file pone.0244341.s009.docx]

**SUPPLEMENTAL INFORMATION OF MYCOBIOME**

PONE-D-20-38095-R1

**Exposure to Traffic-Related Air Pollution is Associated with Greater Bacterial Diversity in the Lower Respiratory Tract of Children**

Christine Uebel-Niemeier^1^, Patrick H Ryan^1,2,3^, Jaroslaw Meller^1,2^, Nicholas J. Ollberding^2,3^, Atin Adhikari^4^, and Tiina Reponen^1^

^1^Department of Environmental Health, University of Cincinnati, Cincinnati, OH, USA

^2^Department of Pediatrics, University of Cincinnati, Cincinnati, OH, USA

^3^ Division of Biostatistics and Epidemiology, Cincinnati Children’s Hospital Medical Center, Cincinnati, OH, USA

^4^Jiann-Ping Hsu College of Public Health, Georgia Southern University, Statesboro, Georgia, USA

**Methods**

For fungal qPCR, the universal fungal primers 5.8Fl and 5.8Rl, and probe, 5.8Pl, for the amplification of the internal transcribed spacer (ITS) regions of fungal rDNA were used (1). Extracted DNA from a solution of *Aspergillus versicolor* with a known concentration of spores was used as the standard.

For fungal sequencing, we used the ITS1F-ITS2aR primers for the amplification of the ITS1 region. The ITS fungal rDNA region was selected over the 18s rDNA region because it has been found to be more precise in fungal community analysis (2). The ITS1 region was selected over the ITS2 region because the pair of primers for the amplification of ITS1 have been found to be more selective, producing fewer non-fungal sequences, in addition to producing a higher number of sequences, than the primer pair for the amplification of the ITS2 region (3). Fungal PCR and sequencing were performed by RTL Genomics, a division of Research and Testing Laboratories.

Primer sequences were removed using the methods described in the article. For fungi, reads with less than 50 base pairs were removed to get rid of suspicious low-length reads. Reads with a quality score less than or equal to two, with a maximum expected error rate for the forward or reverse read greater than two, or with a forward or reverse read that contained an ambiguous base were removed. The UNITE ITS database was used as the reference database (4). Sequences were aligned using the AlignSeqs function in the DECIPHER package v2.12.0 (5). A de novo tree was created for fungi using agglomerative clustering of the sequence distance matrix using USEARCH (6).

For fungi, saliva samples were not included if they did not amplify or had <5000 reads after sequencing. Due to low fungal abundance in sputum, sputum samples were included if they amplified and had >400 reads. These samples were treated as pilot samples. Statistical methods are discussed in detail in the full article.

**Results**

Sputum samples from 10 of the participants were included in the fungal analyses. For sputum, 5 participants were in each TRAP exposure group. Of the 10 samples, 60% of participants were female, 50% were asthmatic, and 90% had mothers with education beyond high school. Saliva samples from 8 of the participants were included in the fungal results. For saliva, 4 participants were in each TRAP exposure group. Of the 8 saliva samples, 50% of the participants were female, 37.5% were asthmatic, and 100% had mothers with education beyond high school.

We compared the sputum mycobiome of high and low TRAP-exposed participants, as well as by gender and asthma status. The median ECAT for the high exposure group was 0.40 mg/m^3^ and the median for the low exposure group was 0.29 mg/m^3^. There was an overlap between confidence intervals for all three alpha diversity measures when comparing TRAP exposure groups, genders, and asthma status groups (Suppl. Table 1). There also were no significant differences in beta diversity (Bray-Curtis) between asthma status groups (p=0.35), between TRAP exposure groups (p=0.94), nor between genders (p=0.23), using the Adonis test. The xdc.sevsample test showed a difference in the distribution of major fungal classes between asthma status groups (p=0.009), and between gender (p<0.001), but not between TRAP exposure groups (p=1.0). It should be noted, however, that due to overall low fungal abundance in sputum, we had a very small sample size.

**References**

1. Haugland R, Vesper S, inventorsMethod of identifying and quantifying specific fungi and bacteria. US Patent US6387652B12002.

2. Liu J, Yu Y, Cai Z, Bartlam M, Wang Y. Comparison of ITS and 18S rDNA for estimating fungal diversity using PCR–DGGE. World Journal of Microbiology and Biotechnology. 2015;31(9):1387-95.

3. Mello A, Napoli C, Murat C, Morin E, Marceddu G, Bonfante P. ITS-1 versus ITS-2 pyrosequencing: a comparison of fungal populations in truffle grounds. Mycologia. 2011;103(6):1184-93.

4. Nilsson RH, Larsson K-H, Taylor AFS, Bengtsson-Palme J, Jeppesen TS, Schigel D, et al. The UNITE database for molecular identification of fungi: handling dark taxa and parallel taxonomic classifications. Nucleic acids research. 2018;47(D1):D259-D64.

5. Wright ES. Using DECIPHER v2. 0 to analyze big biological sequence data in R. R Journal. 2016;8(1):1-8.

6. Edgar RC. Search and clustering orders of magnitude faster than BLAST. Bioinformatics. 2010;26(19):2460-1.
